# Killian’s photographs: “Facies dolorosa”, the countenance of pain 

**DOI:** 10.5414/CP202619

**Published:** 2016-05-18

**Authors:** Veronika Luger, Frank Feistle, Gerhard Feistle, Jörg Feistle

**Affiliations:** Dustri-Verlag Dr. Karl Feistle, Munich, Germany

**Keywords:** facies dolorosa, the countenance of pain, Hans Killian, historical review

## Abstract

Abstract. The book “Facies dolorosa” by Hans Killian, first published in 1934 and later in 1956 and 1967 as extended and annotated versions, comprises ~ 70 photographs depicting facial expressions of patients suffering from various diseases. The photographs in black and white are analyzed and annotated by the author with the purpose of providing clinicians, and especially young doctors, with an insight into the medical and scientific value of facial expression of pain in the diagnosis, staging, and prognosis of severe disease. This historical review of a book no longer in print is part of a 2016 commemorative publication marking the 60^th^ anniversary of the publication of the “Facies dolorosa” by Dustri Medical and Scientific Publications, Munich, Germany and Rockledge, USA.

## Introduction 

The book of photographs compiled by the surgeon Dr. Hans Killian with the title “Facies dolorosa” first appeared in G. Thieme-Verlag in 1934. The considerably extended and annotated versions were published some years later by Dustri-Verlag and are the subject of this historical review. 

## About the author 

Hans Killian was born in Freiburg, Germany, on August 5, 1892. After 2 years of studying medicine in Munich, he was called to do military service in World War I from 1914 to 1918. In 1918, he resumed his medical studies, this time in Freiburg, and received his license to practice medicine on April 1, 1922. Hans Killian then followed a career as a surgeon working initially in the Institute of Pharmacology, University of Munich and later in academic/surgical departments in the university hospitals Dusseldorf and Freiburg. He obtained his Doctor in Medicine degree in 1930 with a highly praised thesis on fat embolism and obtained a post as professor for surgical medicine in Freiburg. He was subsequently called into military service again in World War II where he served in Russia as a consultant army surgeon [1, 2]. 

Killian’s fields of interest were varied and covered general surgery, cardiothoracic and trauma surgery, anesthesia, and the use of curare in medicine. He was also interested in photography, nature, and the arts, and even published a compendium of photographs dealing with the breeding habits of butterflies prior to publication of the “Facies dolorosa”. Hans Killian died on March 7, 1982 at the age of 89 years [1, 2, 3]. 

## About the book 

**Figure 1.**
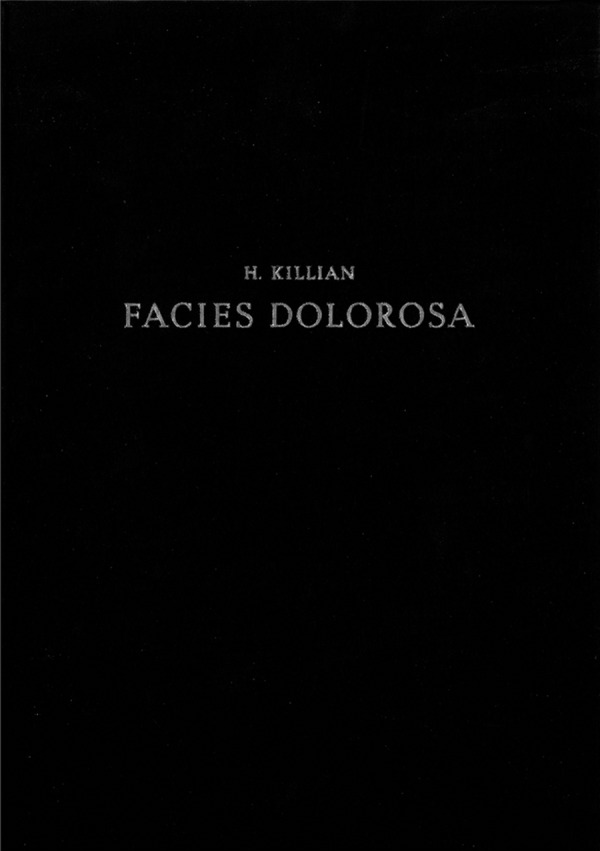
Cover of the 1956 edition.

“Facies dolorosa: Das schmerzensreiche Antlitz” (“The countenance of pain”) was first published in1934 by Georg Thieme, Leipzig [4]. Copies of this first edition, even second hand, are very rare and prices can exceed 1,000 Euros depending on the condition. The first edition comprises 88 pages with 64 photographs. The extended and annotated second edition, published in 1956 by Dustri-Verlag Remscheid-Lennep [5], contains 202 pages and 69 photographs (Figure 1). The third edition was published by Dustri-Verlag Dr. Karl Feistle Munich-Deisenhofen in 1967 and has 156 pages and 73 photographs [6]. 

In the preface to the second edition, the author sets out the aims and purpose of the book and reports his observation that physicians have always strived to diagnose the cause and severity of a patient’s suffering from his or her facial expression. A main purpose of the book with the photographs and annotation therefore was to provide an illustrative and educational text for assisting diagnosis by clinicians and thus therapy and patient care. 

In the introductory chapters, the author describes the history of what he terms general and medical physiognomy, going back to classical antiquity. Through the ages, many scholars and physicians – including Hippocrates (c. 460 – c. 370 BC), Aristotle (c. 380 – c. 320 BC), and Galen (129 – c. 200 AD) – sought to define and classify physiognomic characteristics. They were hoping to learn as much as possible about a person by looking into his or her face, believing that a person’s character or personality could be assessed by the physical appearance, especially the face. In the 1956 edition, Killian criticizes this viewpoint since he is convinced that there is a reciprocal relationship between a person’s current mental state and their facial expression. He does not claim that it is possible to diagnose a certain disease from a person’s face alone, but sets out the hypothesis that facial expression and gesture provide insight into the current phase of a disease and more importantly help to answer the question: “Is the patient getting better or worse ?” 

This is followed by a detailed chapter on the functional basics of facial expression, illustrated with anatomical drawings of the musculature of the human face. Killian describes the different types of facial changes that are anatomically possible and gives examples of diseases where the nature of the disease is reflected in the patient’s face. On the one hand, there are relatively easy-to-diagnose diseases that directly affect the head or face such as tumors or paralysis in this region. Other facial manifestations can be more subtle and less specific such as redness of cheeks in patients with pulmonary tuberculosis. He states that the book should help young doctors, in particular, to become more aware of details such as the color, form, and of course the expression of the face [5]. The author lists a series of facial manifestations that were well known when the book was written, e.g., “Facies febrilis”, “Facies abdominalis”, “Facies asthmatica” etc., and expresses regret that no satisfactory definitions for these manifestations had been put forward. “Facies dolorosa” was thus an attempt to correct this deficiency constituting an atlas of the facial expressions of patients and providing scientific support in the diagnosis and description of diseases [1]. 

The succeeding chapter is a detailed explanation of the photographs, subdivided into: a) Mentally almost unaffected patients; b) How a patient experiences a disease and how this is reflected in his or her face; c) Goiter; d) Anemia; e) Changes of color; f) Facies dolorosa (faces of people suffering from pain due to various reasons); g) Anesthesia; h) Stupor and unconsciousness; i) Cachexia; j) Series of photographs taken at various disease stages. 

**Figure 2.**
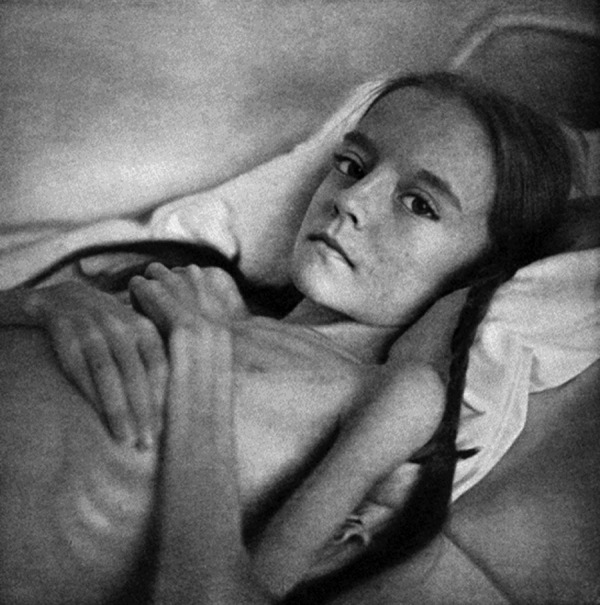
Severe osteomyelitis with metastases and cachexia (Figure 11 in the book).

**Figure 3.**
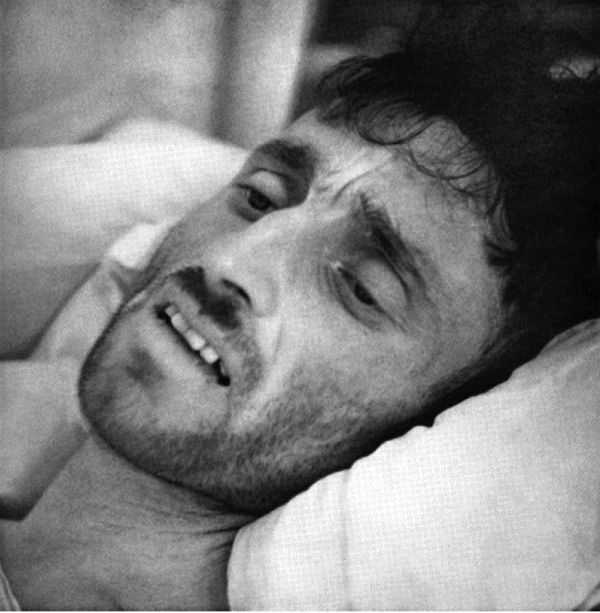
Multiple liver abscesses with icterus after protracted, unoperated appendicitis (Figure 33 in the book).

**Figure 4.**
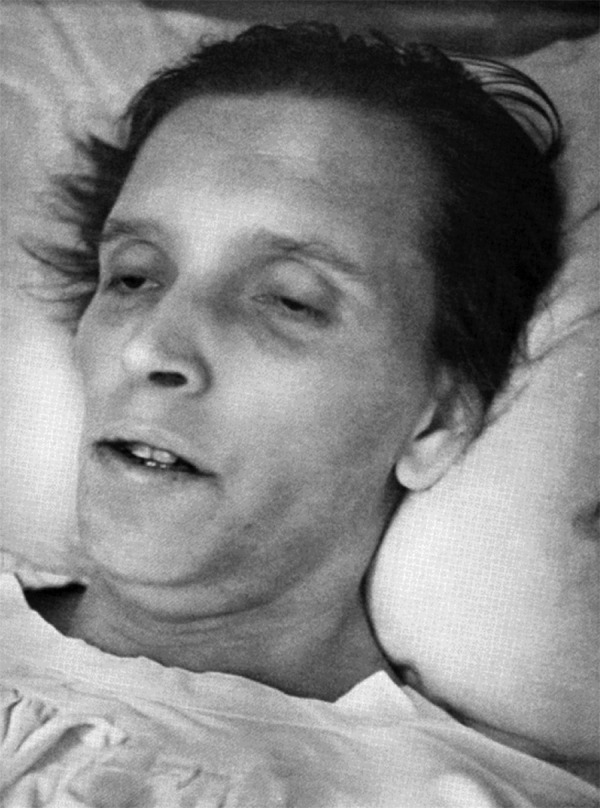
Uremic coma because of bilateral chronic renal suppuration (Figure 44 in the book).

The last chapter comprises the famous photographs taken by Hans Killian himself and the timeless message of the book. A very small selection of these photographs is reproduced here (Figures 2, 3, 4). 

Whereas some information in the book can be considered outdated, Killian’s general message is definitely not. He cites Paracelsus who said a doctor should not only strive for the patient’s physical healing but also for his or her re-entry into the earthly world. In modern words: Not only restoration of the functional abilities should be aimed for, but the physician should also help the patients to get back to their normal lives. Killian concludes with the statement, showing his compassion as a clinician, that nothing can be worse in this context than healing only the body and leaving behind a “mental cadaver”. 

## Acknowledgments 

Dustri-Verlag Archives, Deisenhofen-Munich, Gerrmany. 

## Conflicts of interest 

All authors are associated professionally in various capacities with Dustri Medical and Scientific Publications.
